# Optimizing Nanopore sequencing-based detection of structural variants enables individualized circulating tumor DNA-based disease monitoring in cancer patients

**DOI:** 10.1186/s13073-021-00899-7

**Published:** 2021-05-18

**Authors:** Jose Espejo Valle-Inclan, Christina Stangl, Anouk C. de Jong, Lisanne F. van Dessel, Markus J. van Roosmalen, Jean C. A. Helmijr, Ivo Renkens, Roel Janssen, Sam de Blank, Chris J. de Witte, John W. M. Martens, Maurice P. H. M. Jansen, Martijn P. Lolkema, Wigard P. Kloosterman

**Affiliations:** 1 Department of Genetics, Center for Molecular Medicine, University Medical Center Utrecht and Utrecht University, Utrecht, The Netherlands; 2 Oncode Institute, Utrecht, The Netherlands; 3 Division of Molecular Oncology, Netherlands Cancer Institute, Amsterdam, The Netherlands; 4 Department of Medical Oncology, Erasmus MC Cancer Institute, Rotterdam, The Netherlands; 5 Princess Máxima Center for Pediatric Oncology, Utrecht, The Netherlands; 6 Cyclomics, Utrecht, The Netherlands; 7 Frame Cancer Therapeutics, Amsterdam, The Netherlands

**Keywords:** Genomics, Liquid biopsies, Nanopore, Cancer, Structural variation

## Abstract

Here, we describe a novel approach for rapid discovery of a set of tumor-specific genomic structural variants (SVs), based on a combination of low coverage cancer genome sequencing using Oxford Nanopore with an SV calling and filtering pipeline. We applied the method to tumor samples of high-grade ovarian and prostate cancer patients and validated on average ten somatic SVs per patient with breakpoint-spanning PCR mini-amplicons. These SVs could be quantified in ctDNA samples of patients with metastatic prostate cancer using a digital PCR assay. The results suggest that SV dynamics correlate with and may improve existing treatment-response biomarkers such as PSA.

https://github.com/UMCUGenetics/SHARC.

**Supplementary Information:**

The online version contains supplementary material available at 10.1186/s13073-021-00899-7.

## Background

The detection of cancer recurrence as well as accurate and fast monitoring of response to treatment currently lacks sensitivity for detection of changes over time [1, 2]. Liquid biopsies, which can be used to detect circulating tumor DNA (ctDNA) from body fluids, such as blood, in a minimally invasive manner, are a promising approach to improve monitoring of tumor burden over time [3, 4]. Circulating tumor DNA, which originates from apoptotic and necrotic tumor cells, has been shown to have a positive linear correlation with tumor burden [5]. In multiple cases, ctDNA analyses identified cancer recurrence months before clinical symptoms presented [6–8].

As ctDNA is only a fraction of the total circulating cell-free DNA (cfDNA), it should be distinguished from cfDNA from normal cells by identification of ctDNA-specific genetic alterations. Genomic structural variations (SVs) represent tumor- and ctDNA-specific biomarkers to detect and quantify ctDNA with high sensitivity in liquid biopsies [7–10]. Most solid cancers contain dozens to hundreds of somatic SVs [11, 12]. Besides some recurrent driver SV events that functionally impact tumorigenesis, the vast majority of these somatic SVs are patient- and tumor-specific passenger events [13], which may nevertheless be valuable biomarkers for tumor load tracing. SVs form a unique breakpoint junction between two joined DNA strands and can be validated by straightforward junction-spanning (quantitative) PCR assays, which facilitates their applicability [8].

Somatic SVs are commonly detected with short-read, paired-end next-generation sequencing (NGS). However, as SVs can be very large, short reads are less suited for SV detection [14–16]. Recently, long-read sequencing techniques from Oxford Nanopore Technologies (ONT) and Pacific Biosciences (PacBio) have emerged, and their increased power for germline and somatic SV detection has been extensively demonstrated [15–19]. Moreover, ONT enables a short turnaround time and real-time data analysis [20].

To enable rapid and cost-efficient identification of a set of patient-specific somatic SVs for ctDNA monitoring, we developed a pipeline that leverages the long-read and fast sequencing capabilities of nanopore sequencing in combination with a computational method that enables accurate selection of a subset of somatic SVs from low coverage nanopore sequencing data. The method detects a subset of genomic SVs and can be applied to tumor tissue obtained from (needle) biopsy or resection. The computational approach combines SV calling with random forest classification and germline SV filtering against a blacklist to enrich for somatic SVs without the need of matching germline sequencing data, which reduces the cost and time of the assay. We were able to design SV-specific PCR assays for ctDNA tracking within 3 days after obtaining a tumor biopsy. We validated the pipeline in multiple ovarian and prostate cancer samples. In addition, we demonstrate the clinical applicability of our pipeline by retrospectively tracking the identified somatic SVs in longitudinal cfDNA samples of patients with metastatic prostate cancer, by using digital PCR.

## Methods

### DNA isolation and nanopore sequencing

Several cohorts were used in this study: one melanoma cell line (COLO829), one ovarian cancer organoid line (HGS-3), one cohort of 4 patients with ovarian cancer, and one cohort of 6 patients with prostate cancer.

COLO829 (ATCC® CRL-1974™) cell line was obtained from the American Type Culture Collection (ATCC) and grown according to standard procedures as recommended by ATCC. DNA was isolated using a phenol-chloroform protocol [21]. For some nanopore sequencing runs, DNA was sheared using g-tubes (Covaris). DNA was size selected on the PippinHT (Sage Science). Library preparation was performed using the Lib SQK-LSK109 kit (Oxford Nanopore Technologies), and DNA was then sequenced in 49 separate runs using R9.4 flow cells (Oxford Nanopore Technologies) on the MinION (44), GridION (3), and PromethION (2) instruments (Additional file 1: Table S1).

HGS-3 organoid line was generated from primary patient ovarian cancer tissue at the UMC Utrecht [22] and cultured following the ovarian cancer organoid culture protocol [22]. DNA was isolated by using a phenol-chloroform protocol [21]. DNA was size selected on the PippinHT (Sage Science). Library preparation was performed using the Lib SQK-LSK109 kit (Oxford Nanopore Technologies), and DNA was then sequenced in 40 separate runs using R9.4 (23) and R9.5 (17) flow cells (Oxford Nanopore Technologies) on the MinION (35) and GridION (5) instruments (Additional file 1: Table S1).

Tumor DNA from 4 patients with ovarian cancer was obtained in the UMC Utrecht [22] and isolated with the Genomic-tip kit (Qiagen), following the manufacturer’s protocol for tissue samples, similarly to [22]. DNA was prepared for nanopore sequencing with the Lib SQK-LSK109 (Oxford Nanopore Technologies). The library from one tumor sample was loaded on one revD (Ova1) or R9.4 (Ova2-4) flow cell (Oxford Nanopore Technologies). Sequencing was performed on a MinION (Ova2, Ova4) or GridION (Ova1, Ova3) instrument (Oxford Nanopore Technologies) (Additional file 1: Table S1). Lymphocyte DNA for PCR validation assays was isolated from blood with the DNeasy Blood & Tissue Kit (Qiagen). The blood was obtained in the UMC Utrecht.

Tumor and germline DNA from six patients with prostate cancer were obtained in the Erasmus MC (Pros 1 and 3-6) and Franciscus Hospital, Rotterdam (Pros 2) within the CPCT-02 study, from a fresh frozen core needle biopsy of a metastatic lesion and blood, respectively. DNA was isolated on an automated setup with the QiaSymphony according to the supplier’s protocols (DSP DNA Midi kit for blood and DSP DNA Mini kit for tissue). In the context of the CPCT-02 study, WGS was performed by the Hartwig Medical Foundation, Amsterdam, The Netherlands [23]. Residual tumor DNA (80–250 ng) was used for nanopore sequencing. DNA was prepared for nanopore sequencing with the Lib SQK-LSK109 (Oxford Nanopore Technologies). The library from one tumor sample was loaded on one R9.4 (Pros1), revD (Pros2,3), or high-sensitivity research prototype (Pros4-6) flow cell (Oxford Nanopore Technologies). Sequencing was performed on a GridION instrument (Oxford Nanopore Technologies) (Additional file 1: Table S1).

### Illumina sequencing and analysis (COLO829 and HGS-3)

Short-read WGS was obtained for matched tumor and normal DNA from the COLO829 cell line [24] and the HGS-3 organoid line [22].

SV calling was performed by using GRIDSS (v. 2.0.1) [25] in joint calling mode (tumor+reference) for COLO829 and HGS-3 separately. Somatic SV calls were filtered as in [24] (https://github.com/hartwigmedical/pipeline/blob/master/scripts/gridss_somatic_filter.R).

### Benchmarking somatic SV calling from low coverage nanopore sequencing data

Nanopore data from COLO829 was randomly subsampled to 5x sequencing coverage three times independently with Sambamba [26]. SV calling was performed with NanoSV (v. 1.2.4 ) [17] with a 2-read support threshold: Sniffles (v. 1.0.12) [27] with parameters “--report_BND --genotype -s 2” and NanoVar (v. 1.3.8) [28] with default parameters. In all cases, 8 threads were used and computational resources were measured with GNU Time. True and false positives were calculated using the short-read somatic SV callset described above.

### SV calling and filtering pipeline

The SHARC pipeline is available through https://github.com/UMCUGenetics/SHARC.

Mapping is performed in parallel for each FASTQ file by using minimap2 (v. 2.12) [29] with settings “-x map-ont -a –MD.” The reference genome used is version GRCh37. Sorting and merging of BAM files was done by using sambamba (v. 0.6.5) [26]. SV calling was performed by using NanoSV (v. 1.1.2) [17]. Default NanoSV settings were used except a minimum read count of 2 (cluster_count=2) and minimum mapping quality of 20 (min_mapq=20).

VCFs are filtered by using the command ‘awk ‘$7 == "PASS" && $1 !~ /(Y|MT)/ && $5 !~ /(Y|MT):/ && $5 != "<INS>"’’ to select PASS calls and remove insertions and SVs involving chromosomes Y or MT.

VCFs are then annotated with the distance to the closest single repeat element in the reference genome [30, 31], the closest gap element in the reference genome [31], and the closest segmental duplication element in the reference genome [32]. These elements were taken from the UCSC genome browser (http://genome.ucsc.edu/) [31], using the GRCh37/hg19 genome version.

We trained a random forest (RF) model to filter out false-positive SV calls from nanopore data, similarly as previously described [17]. We expanded the selection of input features for the RF, by including read length, SV calling features, and overlap with repeat features in the reference genome (Additional file 1: Table S3). We trained the classifier on the well-characterized NA12878 Genome in a Bottle (GIAB) sample [33–35], for which high-quality germline SV call sets have been obtained by using Illumina [35], PacBio [34], and Nanopore [33] sequencing. The GIAB SV truth set was generated by intersecting these three GIAB SV sets resulting in a set of 1515 germline SVs. We used $$ \raisebox{1ex}{$2$}\!\left/ \!\raisebox{-1ex}{$3$}\right. $$ of the GIAB truth set as a training set and $$ \raisebox{1ex}{$1$}\!\left/ \!\raisebox{-1ex}{$3$}\right. $$ as a test set. We established a precision-recall curve from 100 bootstrapping runs, where the training data were split into 90%-10% train-test subsets. Based on the precision-recall curve, we defined an operating point of 96% precision and 99.5% recall. The final model was then re-trained on the whole training set and tested on the $$ \raisebox{1ex}{$1$}\!\left/ \!\raisebox{-1ex}{$3$}\right. $$ test set. The performance on the test set was 95.1% precision and 99.6% recall, representing an accuracy of 97.2% (Additional file 2: Fig. S5). SV candidates are classified as “true” or “false” based on this RF model.

We set up two databases of SV calls: (i) SharcDB: containing raw NanoSV calls from nanopore sequencing data of 14 samples, 11 of which belong to this study (COLO829, HGS-3, Ova1, Ova2, Ova3, Ova4, Pros1, Pros2, Pros4, Pros5, and Pros6) and three more for which we had SV calls from high coverage nanopore data: COLO829BL (lymphoblastoid cell line, 50x sequencing depth), VCAP (prostate cancer cell line [36]), and the Genome in a Bottle SV calls GIAB [33]. For tests performed with the samples included in this study, the specific sample was excluded from blacklisting with SharcDB; (ii) RefDB: containing germline calls obtained from WGS short-read data of 59 controls: 19 blood controls from patients with ovarian cancer [22], where germline SVs were called with Manta (v. 1.0.3) [37] with default parameters and 40 healthy individuals (biological parents of individuals with congenital abnormalities) [38] where germline SVs were called with Manta (v. 0.29.5) [37] with default parameters.

SV calls from tumor samples are overlapped with those two databases by using VCF-explorer (https://github.com/UMCUGenetics/vcf-explorer).

Only samples classified as “true” by the RF model and that do not overlap with any sample in the databases qualify for primer design.

Primer design for filtered SV calls is automatized by using Primer3 (v. 1.1.4) [39] with a product size range of 30–230 bp.

SVs with a successful primer design are ranked based on SV length, and the 20 largest are selected for PCR validation. Insertions are filtered out early in the pipeline since the inserted sequence cannot be accurately inferred from the low coverage nanopore sequencing data. Inter-chromosomal translocations are not present in the Top20 ranked SVs because the final ranking is based on SV size and this cannot be determined for inter-chromosomal SVs. However, they are available in the final VCF file and primers are designed by default, so they can be manually selected for PCR validation and assay development.

### Breakpoint PCR

To validate SVs, breakpoint PCR with AmpliTaqGold (Applied Biosystems) was performed according to the manufacturer’s protocol. Ten nanograms of primary tumor DNA (somatic) and 10 ng lymphocyte DNA (germline) per primer pair were used as input. PCR products were loaded and visualized on a 2% agarose gel.

### cfDNA isolation

cfDNA was isolated from ascites fluid of Ova2 (ovarian cancer), obtained in the UMC Utrecht, by using the QIAamp Circulating Nucleic Acid Kit (Qiagen) according to the manufacturer’s protocol. Plasma samples from 4 patients with prostate cancer from Erasmus MC were used for this study. Samples were obtained longitudinally during treatment in 3 × 10 mL CellSave preservative tubes (Menarini Silicon Biosystems, Huntingdon Valley, PA, USA) and processed within 96 h as previously described [40] in the Erasmus MC. For patient Pros1, 13 longitudinal cfDNA samples were obtained; for patient Pros4, 9 longitudinal cfDNA samples were obtained; for patient Pros5, 17 longitudinal cfDNA samples were obtained; for patient Pros6, 6 longitudinal cfDNA samples were obtained. Circulating DNA was isolated with the QIAsymphony® DSP Circulating DNA Kit (Qiagen) according to the manufacturer’s protocol with some minor modifications [41]. All cfDNA samples were quantified by Qubit^TM^ fluorometric quantitation (Invitrogen).

### Quantitative PCR

As primer specificity is essential for reliable interpretation of an end-point assay like digital PCR (dPCR), primers for the detection of structural variants were validated by quantitative PCR (qPCR) on whole genome amplified (WGA) tumor and germline DNA. In brief, qPCR was performed by using the CFX96 Touch™ Real-Time PCR Detection System (Bio-Rad Laboratories), and the final reaction mix consisted of 10 μL SensiFAST^TM^ SYBR ® Lo-Rox mix (Bioline), 0.5 μM forward and reverse primers, and 10 ng of WGA DNA and Ultrapure DNas/RNAse free H_2_O to bring up the reaction volume to 20 μL. The Cycle conditions were as follows: 14 cycles of 10s at 95°C and 30s at from 65 to 58°C (touchdown), followed by 20–40 cycles of 10s at 95°C and 30s at 60°C. In addition, a melt curve was generated from 56 to 95°C to assess the generated PCR products. Based on qPCR results, two primer sets for the detection of SVs in each patient were selected for quantification by dPCR. Primer sets were excluded from use with dPCR when one of the following occurred: >1 PCR product, Cq_germline_-Cq_tumor_ <5, and/or Cq_tumor_ > 20.

### DNA sonication and fragment size analysis

To mimic the length of cfDNA and improve DNA molecule partition, WGA DNA of both tumor and germline were sonicated to a peak size of ~150 bp with the S220 Focused-ultrasonicator (Covaris) according to the manufacturer’s protocol. The sonication conditions were as follows: 200–250 ng WGA DNA (concentration determined by Qubit^TM^ fluorometric quantitation) in 50 μL Ultrapure DNas/RNAse free H_2_O, peak incident power 175 W, duty factor 10%, cycles per burst 200, treatment time 280 s, temperature 7°C, and water level 12. After sonication, DNA fragment sizes were analyzed with the High Sensitivity DNA kit (Agilent Technologies) on the Bioanalyzer (Agilent Technologies) and the sample concentration was re-quantified by Qubit^TM^ fluorometric quantitation (Invitrogen, Life Technologies, Carlsbad, CA, USA).

### Design of digital PCR assays for absolute quantification of SVs in cfDNA

To quantify SVs in cfDNA, dPCR was performed. First, the exact position of the breakpoint as determined by nanopore sequencing was validated. We used already available sequenced Illumina data from the CPCT-02 study (Pros1, Pros4, Pros5, and Pros6), but Sanger sequencing of the particular qPCR product could be used as well. To enable quantification of both mutant and wild-type alleles, additional primers for the detection of wild-type upstream (WT-U) allele and wild-type downstream (WT-D) allele of the breakpoint and fluorescent probes for both mutant and wild-type alleles were developed by using the Primer Express Software v3.0 (ThermoFisher) and the online tool Primer3Plus [39]. All primers and fluorescent probes (Additional file 1: Table S4) were ordered from Eurogentec.

### Pre-amplification of cfDNA

To enable sensitive detection of multiple SVs in limited amounts of cfDNA, two SVs per patient were pre-amplified with 0.2–1 ng of cfDNA. Pre-amplified tumor and germline DNA samples were used as respectively positive and negative control. Pre-amplification was performed by using 4 μL of TaqMan™ PreAmp Master Mix (cat.no: 4488593, Life Technologies), 2 μL primer pool (0.25 μM) consisting of SV forward (SV-F) and reverse (SV-R) primers and upstream (WT-U) and downstream (WT-D) wild-type primers, and 2 μL (cf)DNA for a total volume of 8 μL. Pre-amplification cycle conditions were 10 min at 95°C followed by 14 cycles of 15 s at 95°C and 4 min at 60°C and finally pause at 4°C. After the pre-amplification reaction, 72 μL of Ultrapure DNase/RNAse free H_2_O was added to each sample. Next, pre-amplified cfDNA was diluted 40x per 1 ng input, used for the pre-amplification, to prevent overloading of the dPCR chips.

### Absolute quantification of SVs in cfDNA with digital PCR

For the quantification of SVs in (cf)DNA, dPCR was performed with the Naica Crystal PCR system (Stilla Technologies) by using the following optimized reaction mix: 1 μL of diluted pre-amplified (cf)DNA sample, 5.6 μL PerfeCTa Multiplex qPCR ToughMix (Cat.No: 733-2322PQ, Quantabio). 0.25 μM probes (SV^FAM^, WT-U^HEX^, WT-D^CY5^), 0.75 μM of the SV forward (SV-F) and reverse primer (SV-R), 0.25 μM of the WT-U and WT-D primers, and 0.1 μM Fluorescein (Cat.No: 0681-100G, VWR) and Ultrapure DNAse/RNAse free H_2_O to bring up the total volume to 28 μL. Samples were loaded onto Stilla Sapphire chips (Cat.no. C13000, Stilla Technologies), and dPCR was performed with the same cycle conditions as for the primer validation with qPCR. The median number of analyzable droplets was 21,357, inter-quartile range 19,837–22,736. dPCR reactions were optimized with 10 ng sonicated tumor and germline WGA DNA. When an SV could be detected in pre-amplified cfDNA samples, a dPCR of all longitudinal cfDNA samples was performed on 5 ng of stock (no pre-amplification) cfDNA to enable absolute quantification of mutant molecules in plasma.

### Statistics

qPCR experiments were analyzed with Bio-Rad CFX Manager version 3.1. dPCR experiments were analyzed with Crystal Miner™ software, version 2.1.6 (Stilla Technologies). Thresholds for positive fluorescence were determined per primer pair based on positive and negative controls. Variant allele frequency (VAF) was calculated according to the following formula:

number of mutant molecules per μL in chip (as defined by Crystal Miner™ software)/(number of mutant molecules per μL in chip + number of wild-type molecules per μL in chip) × 100%.

The absolute number of mutant molecules per milliliter plasma was calculated as follows: number of mutant molecules per μL in chip × 28 μL (input in chip)/(used eluate/total volume of eluate × volume of plasma used for isolation).

To correct for zero values on a log scale, +1 was counted to every value and axes were corrected with −1. Spearman’s correlation coefficient was calculated for comparisons of VAF based on upstream wild-type allele vs downstream wild-type allele, two replicates, and pre-amplified vs non-pre-amplified cfDNA samples. The corresponding slope was calculated by using linear regression analysis.

## Results

### Detection of somatic structural variations from low coverage nanopore sequencing of tumor biopsies

**Fig. 1:**
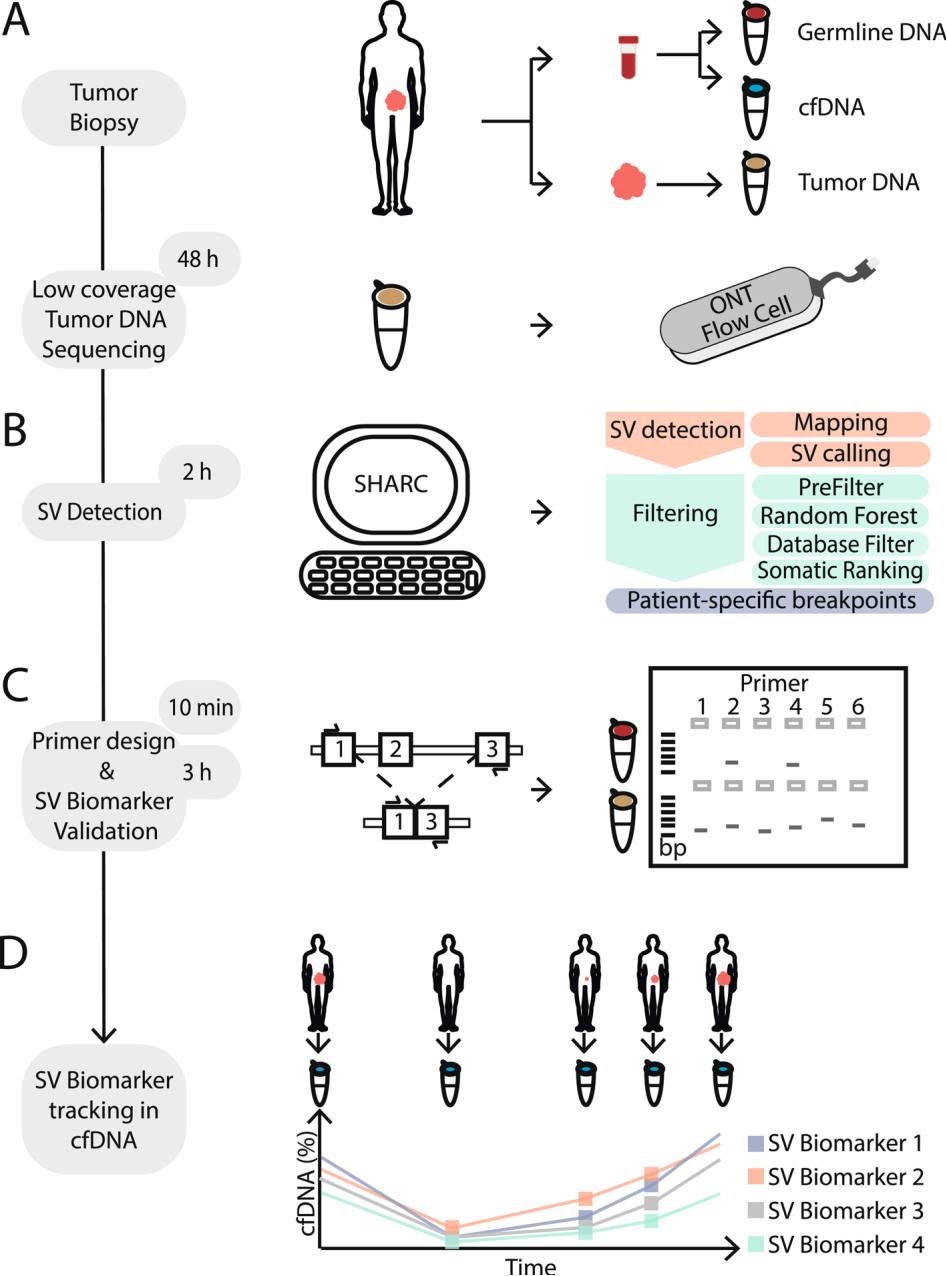
Schematic overview of SHARC. **a** (Needle) biopsy or resection from a tumor as well as blood is obtained from a patient at initial diagnosis. Germline DNA (red) and cfDNA (blue) isolated from blood and tumor DNA (brown) from tumor material. Tumor DNA is sequenced on one ONT flow cell. **b** Tumor-specific SV detection and filtering is performed with the bioinformatic SHARC pipeline. **c** SV-specific breakpoint-spanning primers are designed. Breakpoint PCR with SV-specific primers is performed on germline and tumor DNA to confirm somatic SVs. **d** Somatic SVs are used as biomarkers and traced within cfDNA from a patient to monitor disease dynamics in a longitudinal manner

The first step of our analysis involves low coverage nanopore sequencing of genomic tumor-derived DNA (Fig. 1a). A single nanopore run on the MinION or GridION platforms typically generates between 5–15 Gbs of data [33], corresponding to 1.5–5x coverage of the human genome. Next, the low coverage sequencing data are mapped to the reference genome followed by the detection of SV breakpoint junctions from split read mappings (Fig. 1b) [17]. Subsequently, a classification and filtering pipeline is applied to enrich for somatic SV breakpoints irrespective of corresponding germline data (Fig. 1b). Finally, PCR assays with mini-amplicons are designed to validate the 20 most likely somatic SVs. SVs are confirmed as either somatic or germline by breakpoint PCR on tumor and corresponding lymphocyte DNA (Fig. 1c). Successful breakpoint PCR assays for somatic SVs can then be utilized as biomarkers for ctDNA-based monitoring of treatment response and disease recurrence (Fig. 1d).

### Establishment of a somatic SV reference set

To verify the ability of our pipeline to detect somatic SVs, we used genomic data from the melanoma cell line COLO829 [42] and the ovarian cancer organoid line HGS-3 [22]. We utilized short-read WGS data from both lines (90x and 30x coverage for COLO829 and HGS-3, respectively) and matching reference samples (30x coverage in both cases) to establish two reference sets of somatic SVs (“Methods” section). By using a state-of-the-art somatic SV detection pipeline [43–46], we detected 92 and 295 somatic SVs in COLO829 and HGS-3, respectively. Additionally, we generated long-read nanopore sequencing data for COLO829 and HGS-3, reaching high coverages of 59x (COLO829) and 56x (HGS-3) (Additional file 2: Fig. S1 and Additional file 1: Table S1). To simulate low coverage long-read sequencing of tumor genomes, we randomly subsampled the nanopore sequencing reads to coverages of 4x, 3x, and 2x. The subsampling was performed 20 times independently for each case, to mitigate the effect of chance on the subsampling and subsequent analysis.

**Fig. 2:**
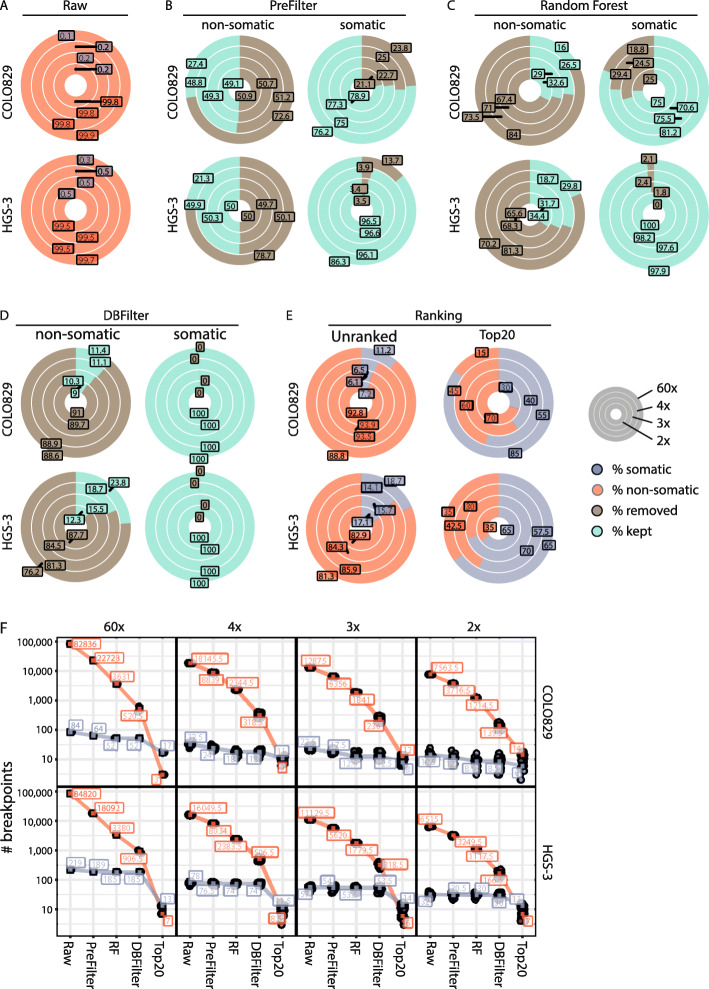
Detection of somatic SVs with the SHARC pipeline based on high and low coverage nanopore data. High coverage nanopore sequencing data from COLO829 (melanoma cell line) and HGS-3 (ovarian cancer organoid) were subsampled to low coverages. Outer circles represent the high coverage sets (59x for COLO829 and 56x for HGS-3) and inner circles represent low coverage subsets (4x, 3x, 2x). The following filtering steps were applied in a cumulative manner in the order displayed. **a** Median percentage of non-somatic (red) and somatic (blue) breakpoints in the raw NanoSV calls for COLO829 (top) and HGS-3 (bottom). **b** Median percentage of non-somatic (left) and somatic (right) SV calls kept (green) or removed (brown) in the pre-filtering step for COLO829 and HGS-3. **c** Median percentage of non-somatic (left) and somatic (right) SV calls kept (green) or removed (brown) by the random forest SV classifier for COLO829 and HGS-3. **d** Median percentage of non-somatic (left) and somatic (right) SV calls kept (green) or removed (brown) by the database filtering for COLO829 and HGS-3. **e** Median percentage of non-somatic (red) and somatic SV (blue) calls in the complete SHARC output (left) and top 20 largest SVs (right) for COLO829 and HGS-3. **f** Total number of non-somatic (red) and somatic (blue) SV calls at each step of the pipeline for both COLO829 and HGS-3. In low coverage subsets, all data points are shown and the square box represents the median value. RF, random forest; DBFilter, database filter

Next, we tested our ability to detect SVs from high and low coverage nanopore sequencing data. First, we compared the performance of the SV callers NanoSV [17, 19], Sniffles [27], and NanoVar [28] to detect somatic SVs in COLO829 data (Additional file 2: Fig. S2). As NanoSV and Sniffles had similar performance with small differences in true- and false-positive rates, we decided to use NanoSV, a previously validated nanopore SV caller [17, 19], to call SVs from the nanopore sequencing data. To maximize sensitivity, we performed SV calling using lenient settings on high and low coverage COLO829 and HGS-3 Nanopore datasets (Additional file 1: Table S2). Based on the overlap with the somatic short-read reference set, raw SV calls were classified as somatic (true positives) or non-somatic (false positives). As expected, the vast majority of the raw SV calls in all the different coverage datasets were non-somatic, on average 99.84% (range 99.81–99.9%, COLO829) and 99.55% (range 99.4–99.74%, HGS-3) (Fig. 2a). In the high coverage Nanopore datasets, we validated 84 (91% of the short-read reference set) and 219 (74% of the short-read reference set) true-positive somatic SVs for COLO829 and HGS-3, respectively, representing a small fraction of the total number of raw SV calls (Fig. 2a and Additional file 2: Fig. S3A). Similarly, we identified an average of 23 (25% of the short-read reference set) and 53 (18% of the short-read reference set) somatic SV breakpoints in each of the low coverage Nanopore sequencing datasets for COLO829 and HGS-3, respectively (Fig. 2a). Altogether, these data underscore that based on lenient SV calling of high and low coverage Nanopore sequencing data with NanoSV, somatic SVs can be identified.

### Enrichment for somatic SV calls from nanopore sequencing data

Since the somatic SVs identified among the SV call sets of the Nanopore data represent only a small fraction of the total raw SV calls, we implemented a panel of cumulative filtering steps to enrich for somatic SVs. Firstly, we selected only “PASS” SV calls (based on default NanoSV filter flags [17], “Methods” section). Secondly, we excluded calls involving chromosome Y or the mitochondrial genome. Finally, we removed all insertions, since the exact inserted sequence cannot be accurately defined from low coverage nanopore sequencing data, thus hampering the final PCR assay development at a later step. As a result of these filtering steps, 72.6% (COLO829) and 76.2% (HGS-3) false-positive calls were removed in the high coverage sets (Fig. 2b and Additional file 1: Table S2). For the low coverage sets, the filtering removed on average 50.9% (COLO829) and 49.9% (HGS-3) of false-positive calls (Fig. 2b and Additional file 1: Table S2). In contrast, the vast majority of true-positive somatic SV calls were maintained following SV filtering (on average 76.9% in COLO829 and 93.9% in HGS-3, Fig. 2b).

To further reduce the number of false-positive SV calls, we employed a random forest (RF) machine learning approach (“Methods” section), similarly as previously described for SV calling of nanopore data [17]. We applied the RF classifier to the filtered high and low coverage subsets of COLO829 and HGS-3. For the high coverage sets, the RF labeled 84% (COLO829) and 81.3% (HGS-3) of false-positive SV calls as false (Fig. 2c). For the low coverage sets, on average, 70.6% (COLO829) and 68% (HGS-3) of false-positive SV calls were labeled as false (Fig. 2c). In addition, in the high coverage sets, 81.25% (COLO829) and 97.88% (HGS-3) of true-positive somatic SV calls were labeled as true. Similar percentages of true-positive SV calls were labeled as true in the low coverage sets, on average 73.7% (COLO829) and 98.6% (HGS-3) (Fig. 2c).

These results show that the RF classifier filters out the majority of non-somatic breakpoints, while maintaining true-positive somatic SV calls. However, germline SV calls are also maintained at this step, requiring further filtering to enrich for somatic SVs (Additional file 2: Fig. S3B).

To reduce the number of germline SVs, we implemented a blacklist filtering step. Therefore, the remaining SV calls were overlapped with two databases (DBFilter) as panel-of-normal (PON) filtering: (i) SharcDB, containing SV calls from nanopore sequencing of 14 different samples, and (ii) RefDB, containing germline SV calls from 59 control samples previously sequenced using Illumina WGS in our group (“Methods” section). Following this filtering step, 100% of true-positive somatic SV calls from both the COLO829 and HGS-3 high and low coverage sets were retained (Fig. 2d). In contrast, 88.6% (COLO829, high coverage), 76.2% (HGS-3, high coverage), and on average 89.9% (COLO829, low coverage) and 84.5% (HGS-3, low coverage) of remaining false-positive SV calls were filtered out (Fig. 2d). Due to this filtering, the fraction of true-positive somatic breakpoints among the remaining SV calls increased to 6.6–18.7%, for the low and high coverage Nanopore datasets of COLO829 and HGS-3 (Fig. 2e and Additional file 2: Fig. S3A).

To further enrich for somatic SVs, we implemented a ranking method, based on the observation that large SVs are more likely to be somatic than germline SVs (Additional file 2: Fig. S4). This increased the percentage of true-positive somatic SVs to 85% (COLO829) and 65% (HGS-3) in the high coverage sets and to on average 43% (COLO829) and 64.1% (HGS-3) in the low coverage sets (Fig. 2e).

Altogether, our SV filtering pipeline strongly enriches for true-positive somatic breakpoints and filters out the majority of false positives and germline SVs. We demonstrate a total enrichment of true-positive somatic SV calls from 0.1% in the raw calls to 85% in the final Top20 ranked calls (17/20, COLO829, high coverage), 0.26 to 65% (13/20, HGS-3, high coverage), on average 0.18 to 41.7% (8.3/20, COLO829, low coverage sets), and on average 0.49 to 64.2% (12.8/20, HGS-3, low coverage sets) (Fig. 2f). Of note, despite low coverage sequencing, each of the somatic SV calls identifies breakpoints at nucleotide resolution, providing immediate access to breakpoint PCR testing.

### Validation in tumor tissue from patients with ovarian and prostate cancer

**Fig. 3:**
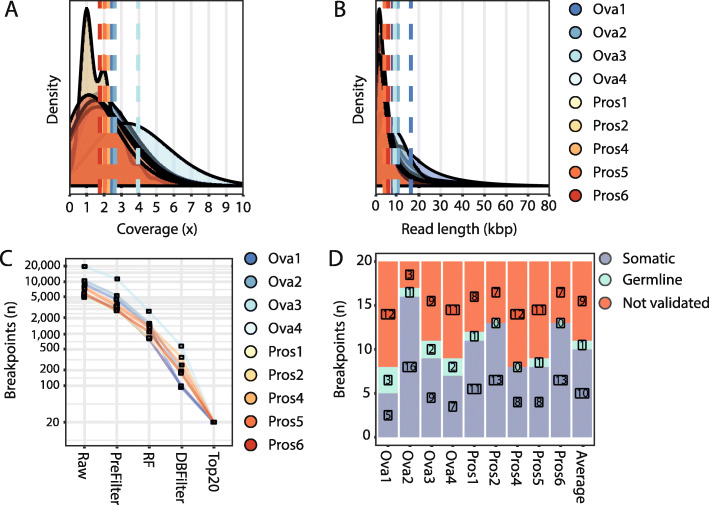
SHARC identifies and validates tumor-specific SV biomarkers from low-pass nanopore tumor sequencing data. Plots showing the distribution of **a** coverage and **b** read length for the nine tumor samples sequenced on one flow cell each. Dashed lines represent averages for each sample. **c** Total number of somatic SVs present at each of the steps throughout the SV calling and filtering pipeline. RF, random forest; DBFilter, database filter. **d** The Top20 ranked breakpoints for each sample were tested by breakpoint PCR using tumor and germline DNA. The graph depicts the number of breakpoints validated as somatic (blue), germline (green), or breakpoints that could not be validated (red)

Next, we tested the pipeline on four high-grade serous ovarian cancer (Ova1-4) and six prostate cancer (Pros1-6) samples. We sequenced tumor DNA on one nanopore flow cell per sample. The ovarian cancer samples and three prostate cancer samples (Pros1-3) were sequenced on commercial ONT flow cells. For the ovarian cancer samples, we started library preparation with minimally 1 μg of DNA. For the prostate cancer samples, limited material was available, and we started library preparation with 250 ng of DNA. For one sample (Pros3), not enough sequencing data was produced to confidently detect somatic SVs and this sample was therefore excluded from all subsequent analyses (Additional file 1: Table S1). Three additional prostate cancer samples (Pros4-6) were sequenced on ONT research prototype flow cells with higher sequencing sensitivity, thus requiring less DNA input material. In these cases, library preparation was started with an average of 108 ng (80–128 ng) of DNA and an average of 10 ng of library was loaded for sequencing (Additional file 1: Table S1**)**. We obtained an average sequence coverage of 2.3x (range 1.8–4.0) (Fig. 3a and Additional file 1: Table S1) and average read lengths of 7.8 Kbp (range 4.2–16.3 Kbp) (Fig. 3b and Additional file 1: Table S1). The sequencing throughput was not affected by the lower DNA input when using the high-sensitivity prototype flow cells (Additional file 1: Table S1).

Following the lenient SV calling, pre-filtering, RF classification, database filtering, and ranking steps, an average of 2.8% (range of 1.0–4.4%) of SVs per sample were retained (Fig. 3c). We performed breakpoint PCR assays on lymphocyte and tumor DNA for the Top20 ranked SVs and validated an average of 10 (50%, range 25–80%) somatic SVs per sample (Fig. 3d). Therefore, despite not having enough sequencing depth to provide a complete genome construction, we were able to identify several somatic SV biomarkers in each of the tumor samples. It should be noted that the annotated ranked VCF with all the breakpoints, prior to Top20 selection, is also reported in case the user wants to manually select other breakpoints and their corresponding primers for validation.

We investigated the recall of validated somatic SVs at different timepoints during the sequencing run. We found that, on average, 81.6% (range 50–100%) of validated somatic SVs were already detected within the first 24 h of sequencing (Additional file 2: Fig. S6). This offers the opportunity to reduce the sequencing time, accelerating tumor biomarker discovery with 1 day.

### Detection of somatic SVs in cfDNA from patients with ovarian and prostate cancer

**Fig. 4:**
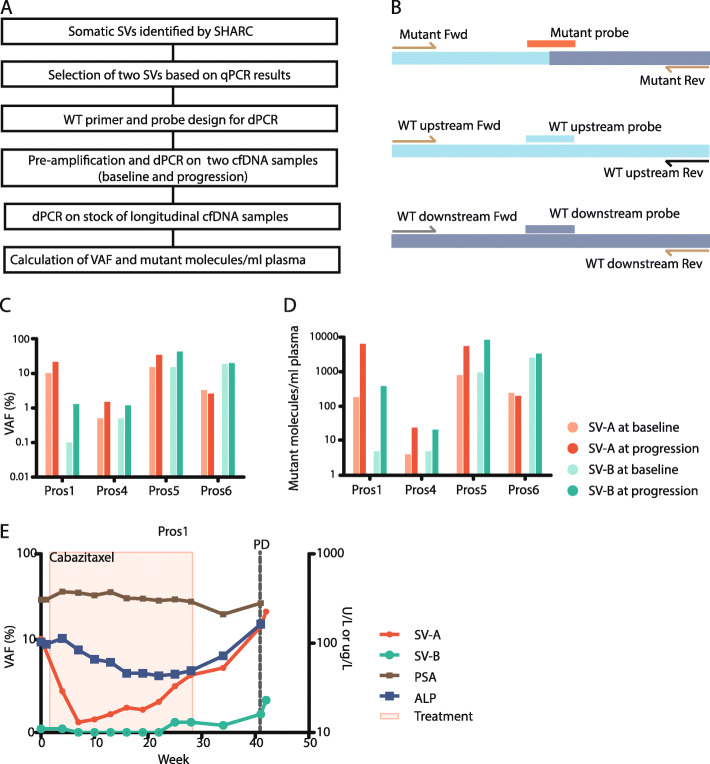
dPCR-based quantification of SVs in blood. **a** Schematic overview of quantification of tumor-specific SVs, identified by SHARC, in cfDNA from blood by using qPCR and dPCR. **b** Primer and probe design for dPCR. The wild-type upstream and wild-type downstream alleles share each one primer with the mutant allele. Three probes with different fluorescents were designed to specifically detect the mutant allele or one of the wild-type alleles. **c** Detection of two tumor-specific SVs in cfDNA from blood from four patients with prostate cancer at baseline and at the progression of disease with dPCR. Shown are VAF and **d** mutant molecules per milliliter plasma. **e** Quantification of SVs in longitudinal cfDNA samples from blood of patient Pros1. The graph depicts VAFs of SVs, treatment, laboratory parameters (prostate-specific membrane antigen (PSA), alkaline phosphatase (ALP)), and clinical progression of disease (PD)

To show the applicability of the pipeline to detect clinically relevant biomarkers, we next tested if we could detect the validated somatic SVs in cfDNA of patients. Ascites fluid, which is known to contain cfDNA and ctDNA [47], was available for Ova2 at the time of disease recurrence. We extracted cfDNA from the ascites and tested the 16 validated somatic SVs out of the Top20 by PCR. One hundred percent of somatic SVs could be detected within the cfDNA from ascites (Additional file 2: Fig. S7), and not in the germline or water controls. Next, we tested whether validated SVs could be detected in cfDNA from blood. Therefore, we selected two patient-specific SVs for four prostate cancer patients (Pros1, 4, 5, and 6) based on a high signal to noise ratio observed in qPCR assays for SV breakpoints (Fig. 4a and Methods).

To enable sensitive and quantitative detection, we designed digital PCR (dPCR) assays for the eight selected SVs (Fig. 4b). For each SV, we aimed to design a probe for both wild-type alleles (up- and downstream) and for the mutant allele (across the breakpoint junction). For five SVs, we could design an assay that quantified both the upstream and downstream wild-type allele. For the three other SVs, primers/probes for only one of the wild-type alleles were designed, as appropriate primer design for the other allele was hindered by repetitive sequences at the target site. As the amount of cfDNA within one liquid biopsy is limited, we used a conditional breakpoint detection approach: (i) if dPCR on pre-amplified cfDNA (input pre-amplification, 0.2–1 ng cfDNA) confirmed the presence of the SV within cfDNA and (ii) then subsequent dPCR on non-pre-amplified cfDNA (stock cfDNA) (input dPCR, 5 ng cfDNA) was performed. The latter enabled calculation of both the variant allele frequency (VAF) and the number of mutant molecules per milliliter plasma (MM/mL plasma). First, we selected two timepoints per patient, one at baseline and one at the progression of the disease, and confirmed the presence of all eight SVs with dPCR on pre-amplified cfDNA (Additional file 2: Fig. S8). Thereafter, dPCR on the stock cfDNA successfully detected all SVs in the four patients, both in baseline and progression samples (Fig. 4c, d). Despite the fact that the VAF in pre-amplified cfDNA correlates to the VAF in stock cfDNA (*r*_s_ = 0.928), they should be considered two separate outcome measurements (regression coefficient = 0.72 ≠ 1) (Additional file 2: Fig. S9A). Moreover, VAF based on the wild-type upstream allele was highly similar to VAF based on the wild-type downstream allele in stock cfDNA (*r*_s_ = 0.996, regression coefficient = 1.05) (Additional file 2: Fig. S9B), suggesting no significant imbalances between the two sides of the breakpoint.

### Monitoring treatment response in patients with prostate cancer

In addition to the detection of SVs in cfDNA at baseline and progression of the disease, we explored the capacity to use SVs to monitor treatment response over time. To enable reliable response monitoring, measurements should be accurate and repeatable. As VAFs are ratios and in principle not influenced by technical variations between timepoints, we chose to report VAFs only. To verify the accuracy of dPCR, we performed two technical replicates for all pre-amplified samples of Pros5 and Pros6 and confirmed a high correlation of VAFs between the replicates (*r*_s_ = 0.987, regression coefficient = 0.918) (Additional file 2: Fig. S9C). Finally, we quantified the eight SVs of the four prostate cancer patients in the longitudinally collected samples from before, during, and after treatment. For Pros1, SV-A shows the potential to improve response evaluation as its dynamics correspond to the expected response to treatment with cabazitaxel and increases towards the end of treatment, resulting in the highest levels at clinical progression of disease (Fig. 4e). These changes also seem to correlate with other blood biomarkers, including prostate-specific membrane antigen (PSA) and alkaline phosphatase (ALP). In addition, SV-B in Pros1 similarly correlates with response to treatment (Fig. 4e). Also, for Pros5, both SV-A and SV-B show clear changes over time correlating with clinical parameters, and Pros4 and Pros6 have less compelling dynamics of the detected SVs (Additional file 2: Fig. S10A-C).

## Discussion

Recent studies have utilized somatic SVs for tracking tumor burden from liquid biopsies [7–10]. Although these studies showed the potential of this methodology, they lacked sufficient turnaround time to provide personalized biomarkers before the initiation of patient treatment. This is due to lengthy short-read WGS approaches for SV detection and an associated substantial number of false-positive somatic SVs, requiring laborious testing to validate SVs. To overcome these limitations, we utilized the real-time and long-read capabilities of nanopore sequencing combined with a machine learning approach to efficiently identify a set of somatic SVs from tumor tissue within 3 days. The rapid and simple workflow offers great potential for routine monitoring of cancer dynamics. We illustrate the applicability of our method to measure tumor burden by using a series of longitudinally gathered blood samples from metastatic prostate cancer patients.

Obtaining enough tumor material for DNA isolation is often a limiting factor for next-generation sequencing assays. We show that nanopore sequencing and somatic SV detection are possible from limited amounts of DNA that can be extracted from a metastatic tumor needle biopsy, which is an important requisite for clinical viability. DNA input can be decreased even further to as little as 80 ng when using flow cells with increased sensitivity for DNA (research prototype flow cells provided by ONT).

Long-read sequencing is an excellent method for the detection of SVs at nucleotide resolution, even at low sequencing depth, because each long read that bridges a breakpoint junction provides direct information on the breakpoint position and sequence [17]. Sequencing of a tumor sample on a single GridION/MinION nanopore flow cell generates insufficient sequencing data to accurately establish a complete genomic profile. However, using the pipeline developed here, we efficiently enriched for patient-specific somatic SV events—irrespective of their functional impact on tumor biology. Despite the very low coverage, the computational method functions independently of corresponding germline sequencing data. These assets make our pipeline a cost-efficient assay for the detection of personalized somatic SV biomarkers. Furthermore, on average, 50% of the detected SVs are somatic, which minimizes the hands-on effort needed for validation purposes. For all analyzed tumors, we identified at least five somatic SV biomarkers per patient, an amount within the range of biomarkers used to trace ctDNA in previous work [7, 9, 48]. With expected increases in sequencing throughput from ONT sequencing, the performance of the pipeline will improve significantly. Furthermore, the use of cheap disposable flow cells (Flongle) could reduce assay costs to $$ \raisebox{1ex}{$1$}\!\left/ \!\raisebox{-1ex}{$5$}\right. $$ of the current sequencing price of 800€ [49]. The minimal costs of this assay would enable the broader application of such individualized SV monitoring in cancer patients.

We retrospectively traced levels of ctDNA with two SVs per patient for four prostate cancer patients and compared tumor dynamics to clinical biomarkers such as PSA and ALP. The quantitative measurement of SVs in ctDNA suggests that VAFs of SVs correlate with tumor load (Pros1 and Pros5). Moreover, the SVs would have indicated progression of disease earlier than PSA did in some patients (Pros1 and Pros 4). Even though we only tested two SVs per patient, this clearly illustrates the potential clinical utility of quantifying ctDNA with SVs to monitor response to treatment. The assay could be optimized by not only identifying the tumor-specific SVs, but also SVs that represent the dominant disease clone and upcoming, targetable subclones. In addition, larger prospective studies should confirm that indeed measuring SVs improves clinical decision-making in patients with metastatic prostate and other cancer types.

## Conclusions

Clinicians are well aware of the dynamic response of cancer to treatment but lack the tools to monitor these changes in real time and thus generally respond to alterations too late for true treatment success. We present a method to overcome these limitations and provide a solution to immediate individualized disease monitoring. This approach could increase the sensitivity of disease monitoring to such levels that more intelligent treatment approaches could be envisioned.

## Supplementary Information

**Additional file 1: Supplementary Tables**.

**Additional file 2: Supplementary Figures**.

## Data Availability

Nanopore sequencing data is available at the European Nucleotide Archive (ENA) and through controlled access at the European Genome-phenome Archive (EGA) as follows: • COLO829 cell line: ENA accession ERX2765498 (https://www.ebi.ac.uk/ena/data/view/ERX2765498) [50] • HGS-3 organoid line: EGA dataset accession EGAD00001005476 (https://ega-archive.org/datasets/EGAD00001005476) [51] • Ovarian and prostate tumor material: EGA study accession EGAS00001003963 (https://ega-archive.org/studies/EGAS00001003963) [52] Data access requests will be evaluated by the UMCU Department of Genetics Data Access Board (EGAC00001000432, https://ega-archive.org/dacs/EGAC00001000432). SHARC SV filtering pipeline is available through https://github.com/UMCUGenetics/SHARC [53] and Zenodo [54].

## Abbreviations

ALP: Alkaline phosphatase
cfDNA: Cell-free DNA
ctDNA: Circulating tumor DNA
dPCR: Digital PCR
GIAB: Genome in a Bottle
NGS: Next-generation sequencing
OC: Ovarian cancer
ONT: Oxford Nanopore Technologies
PacBio: Pacific Biosciences
PC: Prostate cancer
PD: Progression of disease
PON: Panel-of-normal
PSA: Prostate-specific membrane antigen
qPCR: Quantitative PCR
RF: Random forest
SV: Structural variant
VAF: Variant allele frequency
WGA: Whole genome amplified
WGS: Whole genome sequencing
WT-D: Wild-type downstream
WT-U: Wild-type upstream
