# Supplementary material for: Optimizing Nanopore sequencing-based detection of structural variants enables individualized circulating tumor DNA-based disease monitoring in cancer patients
Source: Genome Med. 2021 May 18;13:86. doi: 10.1186/s13073-021-00899-7 (PMC8130429; doi:10.1186/s13073-021-00899-7)
Supplement: Supplementary file 2 — Additional file 2: Supplementary Figures. [file 13073_2021_899_MOESM2_ESM.docx]

**Supplementary figures to:**

**Optimizing Nanopore sequencing-based detection of structural variants enables individualized circulating tumor DNA-based disease monitoring in cancer patients**

**
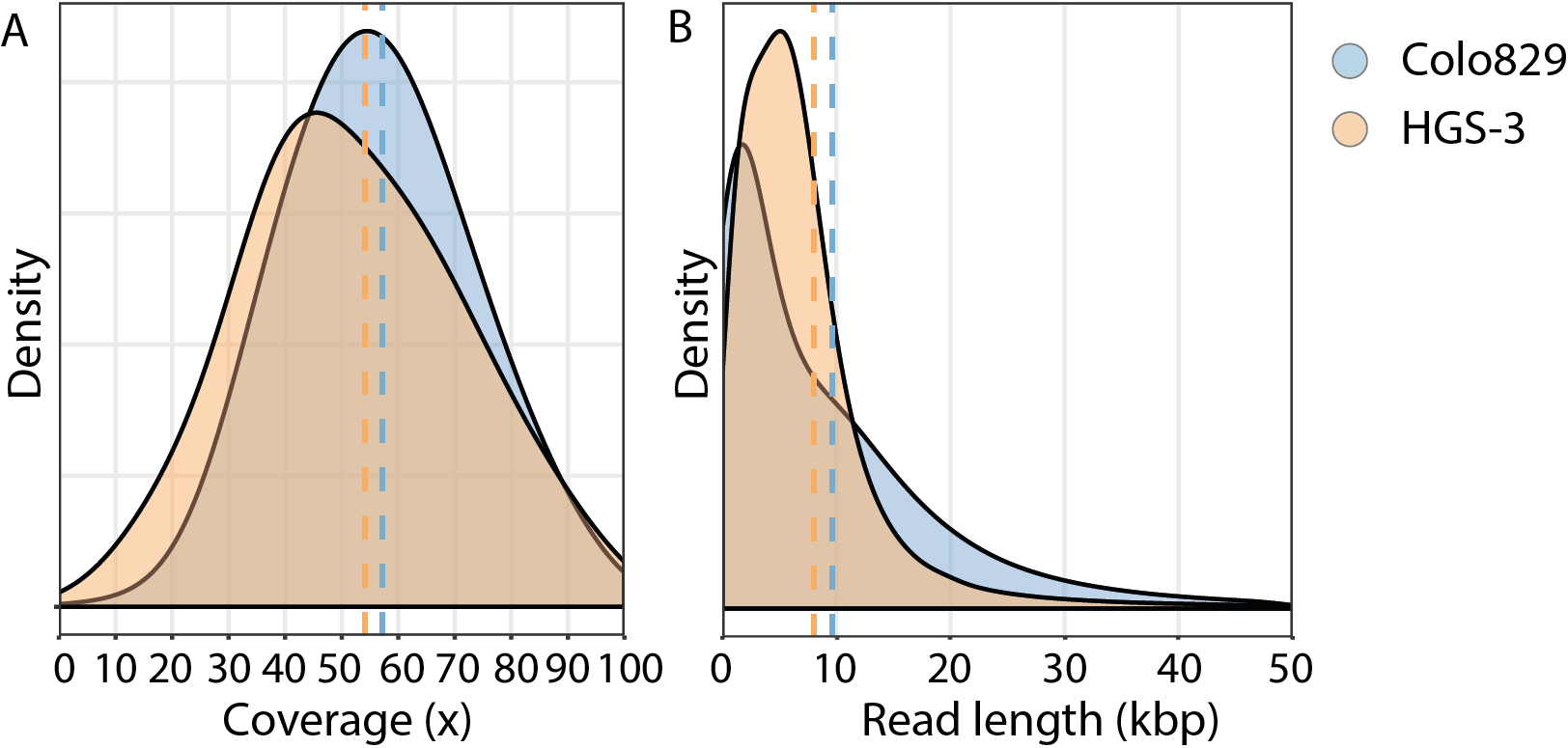
**

**Figure S1: Coverage and read length of COLO829 and HGS-3** Coverage (**A)** and read length (**B**) distribution for COLO829 and HGS-3 nanopore sequencing data. Dashed lines represent average.


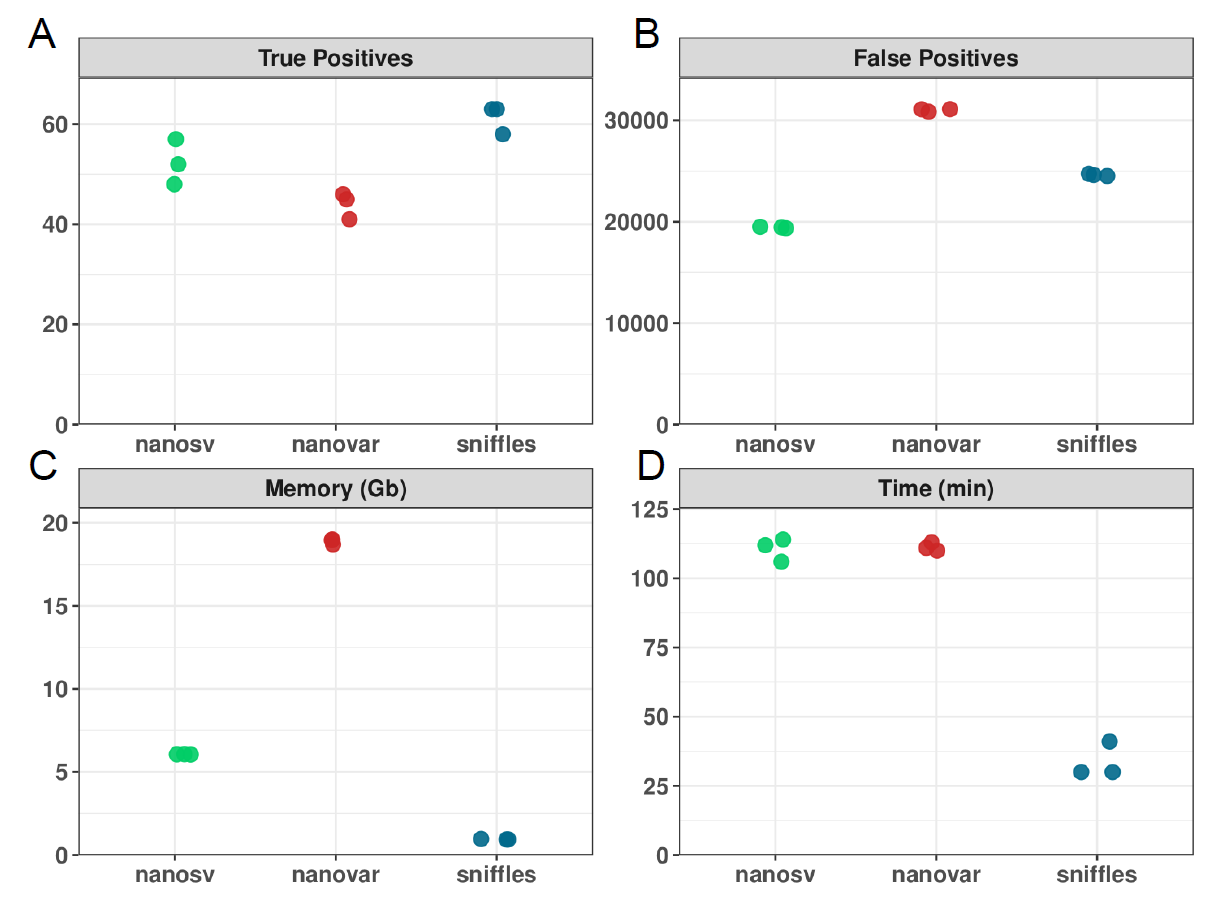


**Figure S2**: **Benchmarking of nanopore SV callers on low coverage nanopore sequencing data.** The SV callers NanoSV, Sniffles and NanoVar are compared in terms of true positives (**A**), false positives (**B**) and required computation memory (**C**) and time (**D**). Triplicates of 5x randomly subsampled COLO829 data were used, and comparisons were performed against a short-read somatic SV reference set.

**
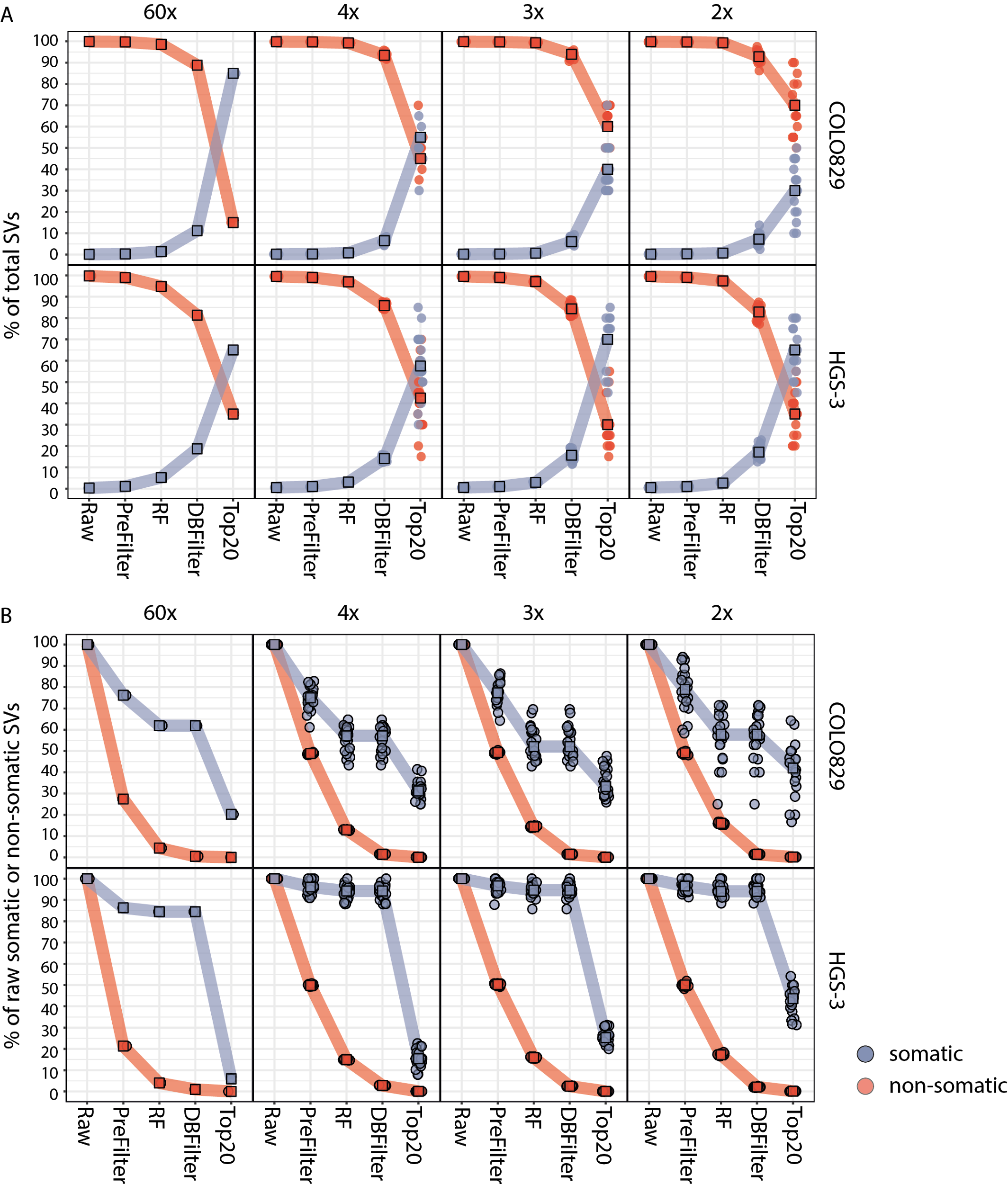
**

**Figure S3: Enrichment of somatic SV calls of COLO829 and HGS-3 after subsequent steps of the SHARC pipeline.** The filtering steps were applied in a cumulative manner in the order displayed for subsampled Nanopore sequencing datasets from COLO829 and HGS-3. For each level of coverage, 20 independent subsampled datasets were generated and subjected to each filtering step in a cumulative manner. (A) Enrichment for somatic SVs after subsequent steps of the SHARC filtering pipeline. The blue and red lines/dots indicate the percentage of somatic and non-somatic SV calls after each filtering step of the pipeline for both COLO829 and HGS-3. The percentage of somatic and non-somatic SV calls is calculated relative to the sum of remaining somatic and non-somatic SV calls after each filtering step. Thus, 100% represents the total number of SV calls (somatic plus non-somatic) present at each step. (B) This figure panel is based on the same underlying data as for panel A, but here the percentage of somatic (blue) and non-somatic (red) SV calls is plotted relative to the total number of somatic and non-somatic SV calls detected at the first step, respectively. Thus, 100% represents the total number of non-somatic or somatic SV calls found initially in the raw data prior to filtering. While the percentage of non-somatic SV calls (red line/dots) decreases rapidly to very low percentages, the percentage of true positive somatic SV calls (blue line/dots) remains substantial (around 20%, depending on the sequence coverage). In low coverage subsets, all data points are shown and the square box represents the median value. RF: Random forest; DBFilter: Database filter.

***
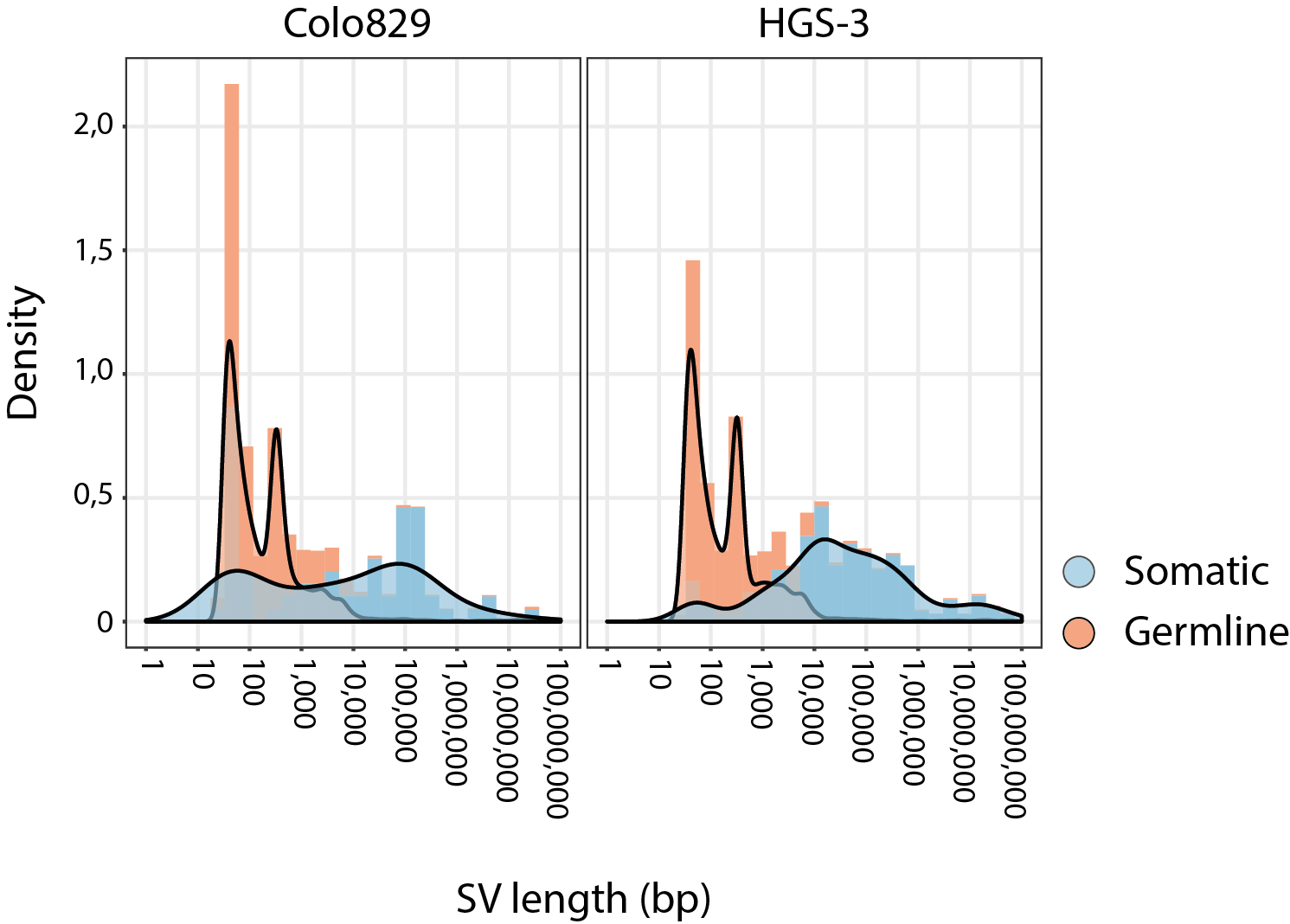
***

**Figure S4: Somatic vs germline SV length** Histogram and density plot of SV lengths of somatic and germline SVs from short-read data of COLO829 and HGS-3.

**
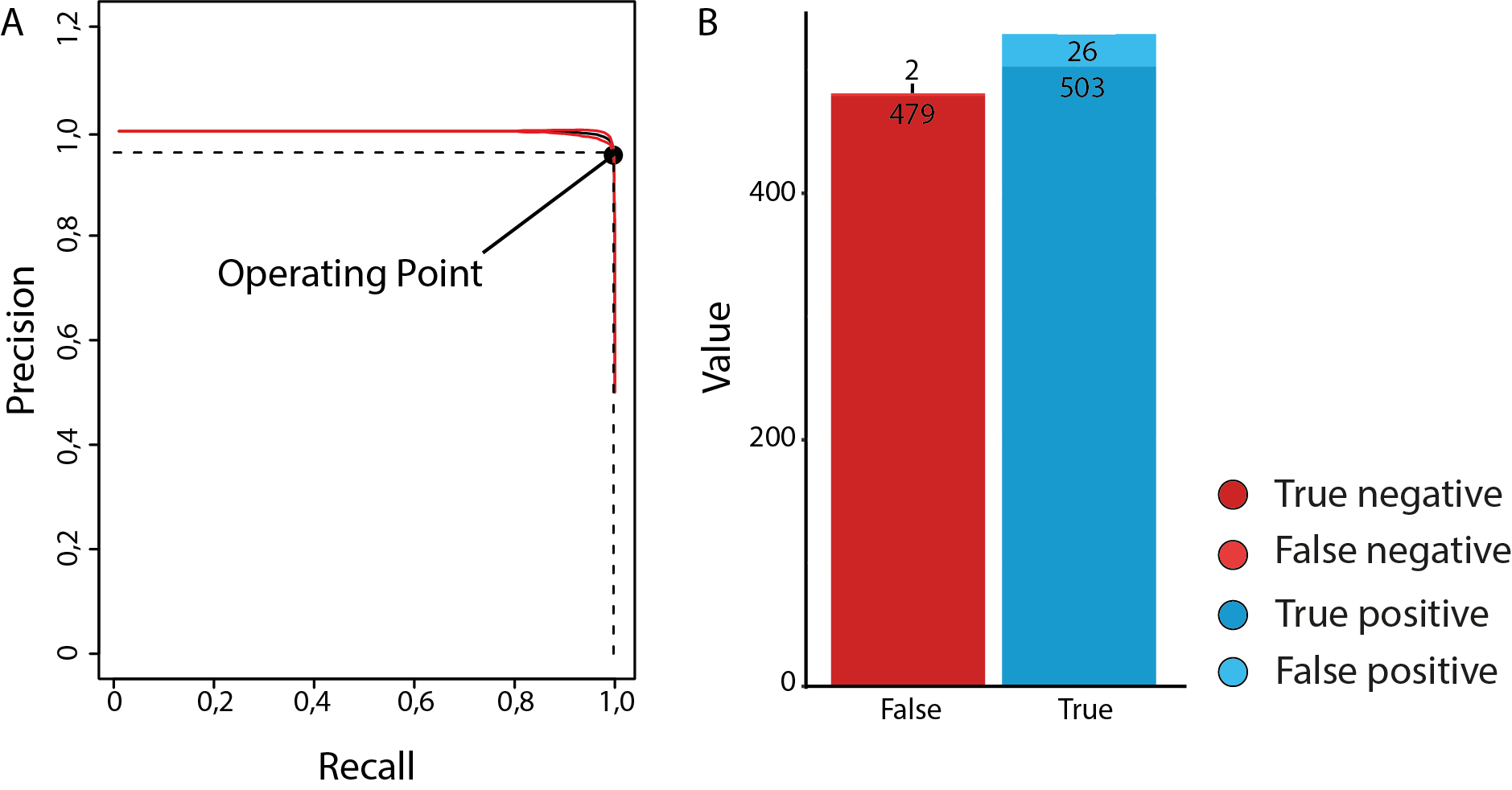
**

**Figure S5: Random forest performance on the Genome in a Bottle sample (GIAB)** (**A**) Precision vs recall curve on the training set. Depicted is the operating point selected of 96% precision and 99.5% recall. (**B**) Random forest performance on the hold-out set.

**
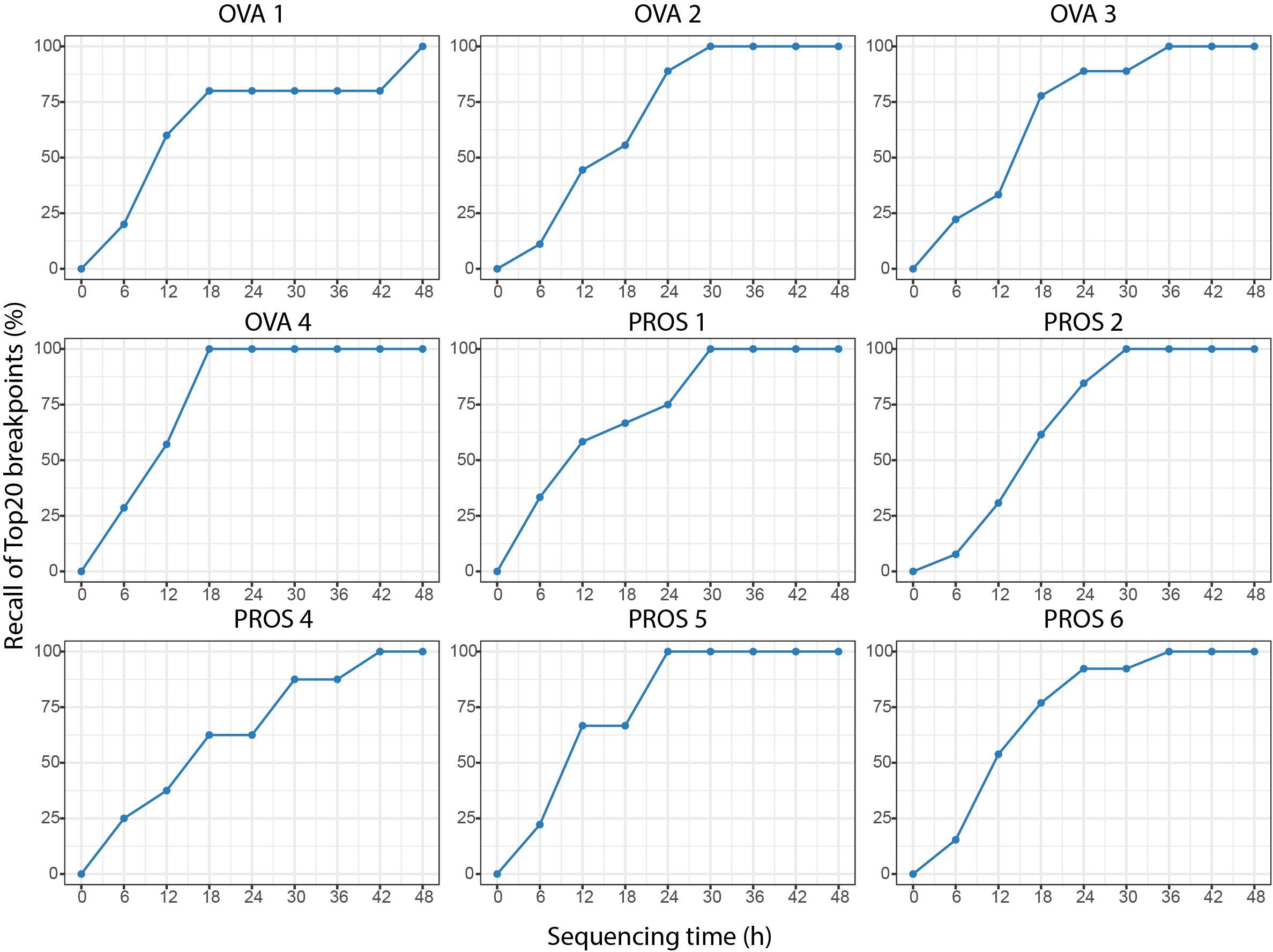
**

**Figure S6: Nanopore sequencing time vs. somatic SV detection** Plots showing the sequencing time and the recall of validated somatic SVs in 6-hour cumulative bins.

**
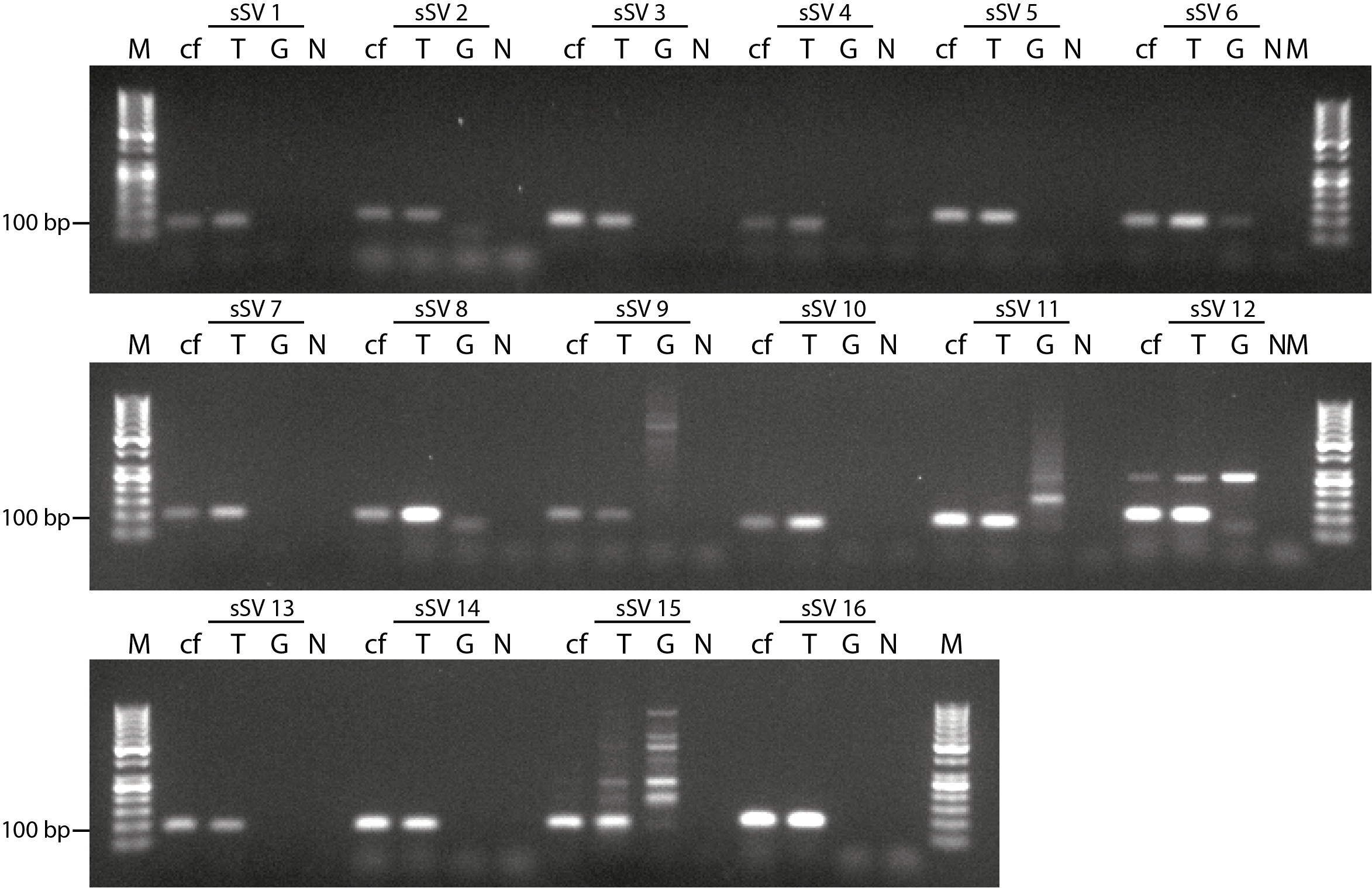
**

**Figure S7: Validation of somatic SV (sSV) of Ova2 biomarkers in cfDNA** sSVs of patient Ova2 were tested on cfDNA from ascites (cfDNA), tumor DNA (T), germline DNA (G) and water control (N). M = DNA ladder

**
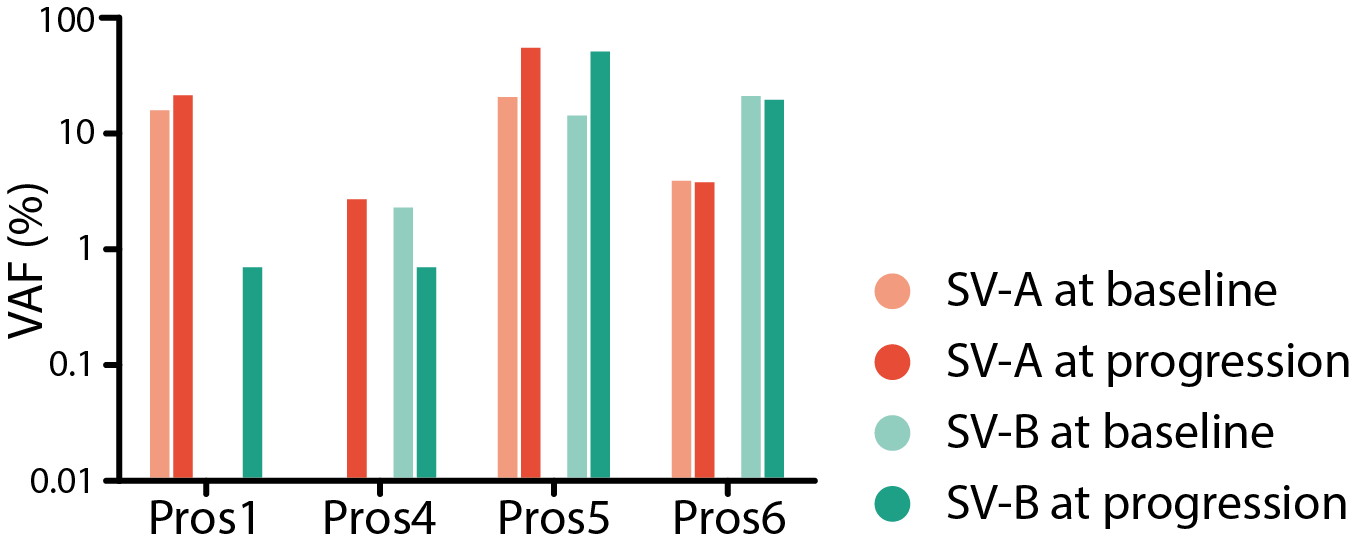
**

**Figure S8: Confirmation of presence of SVs in pre-amplified cfDNA** Detection of two patient-specific SVs in cfDNA from blood from four prostate cancer patients at baseline and at progression of disease with dPCR. Shown are VAFs.

**
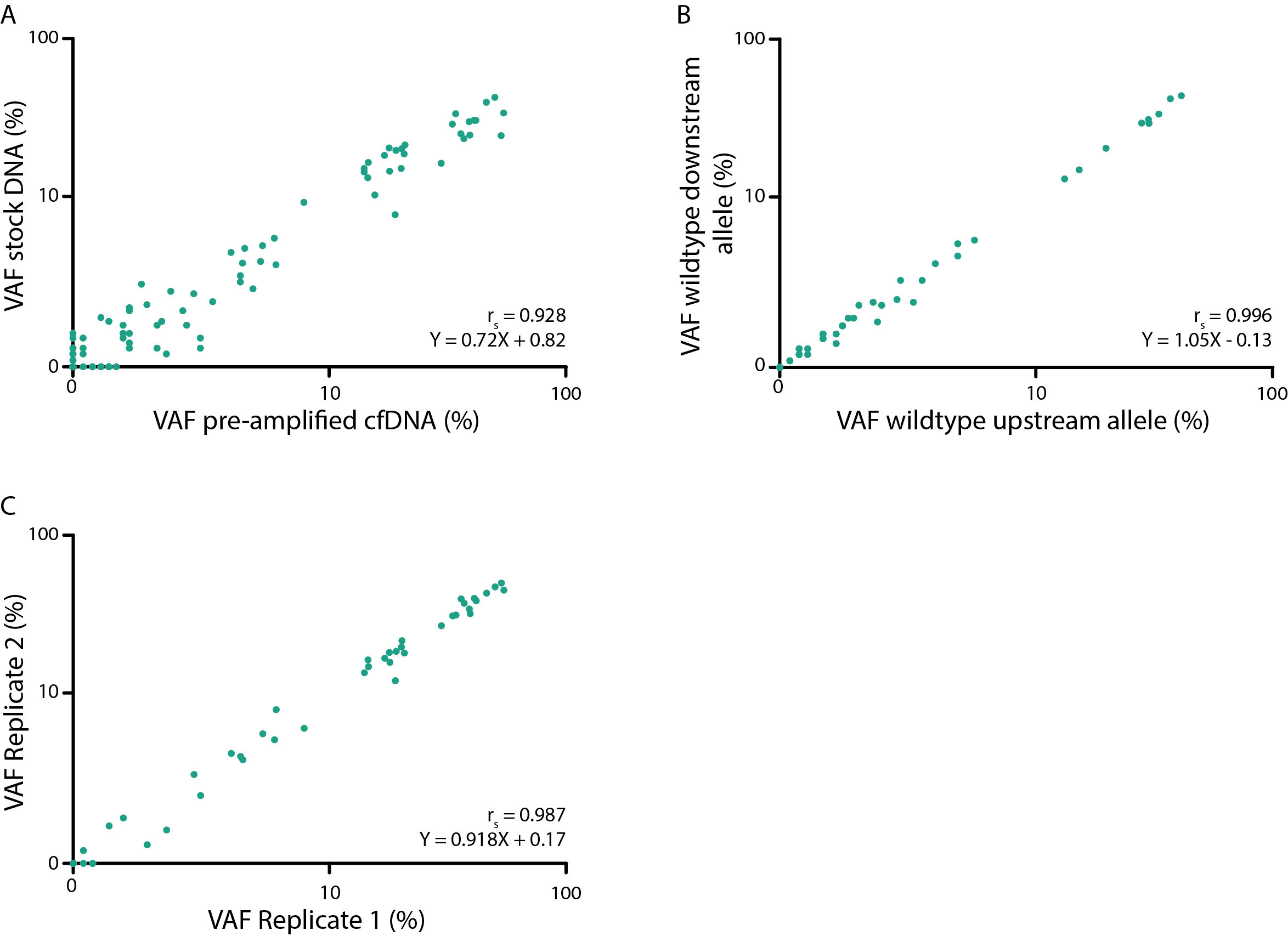
**

**Figure S9: Technical aspects of dPCR** (**A**) Comparison of VAF in pre-amplified cfDNA and VAF in stock (non-pre-amplified) cfDNA. (**B**) Comparison of VAF based on up- and downstream wild type alleles. (**C**) Comparison of VAF in technical replicates of dPCR of pre-amplified cfDNA samples of Pros5 and Pros6.

**
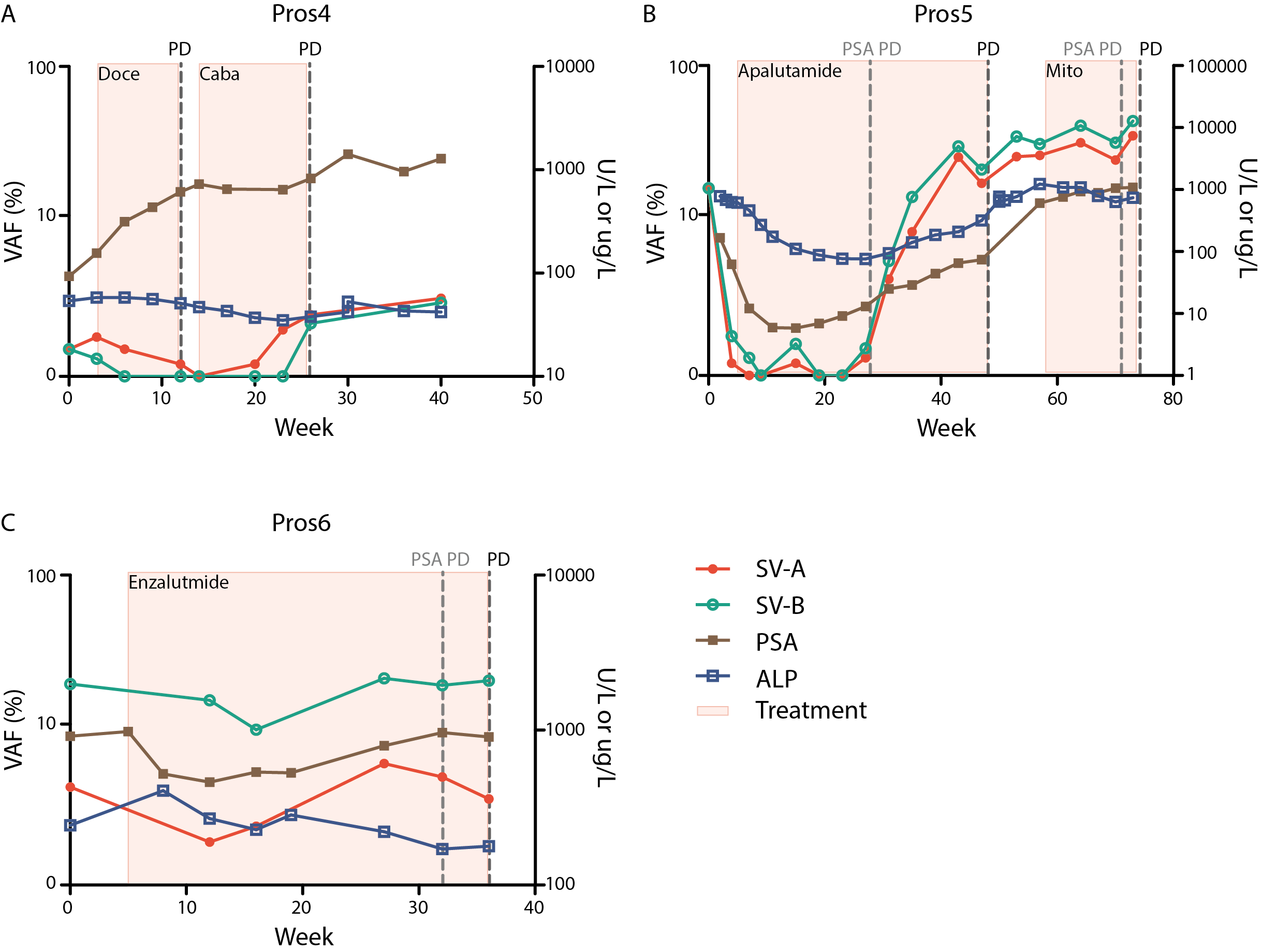
**

**Figure S10: dPCR-based quantification of SVs in blood** Quantification of SVs in longitudinal cfDNA samples from blood in patient (**A**) Pros4, (**B**) Pros5 and (**C**) Pros6. In addition to VAFs of SVs, treatment, laboratory parameters (prostate specific membrane antigen (PSA), alkaline phosphatase (ALP)) and clinical progression of disease (PD) are visualized. Progression of disease based on a confirmed increase of prostate specific membrane antigen (PSA) of ≥25% above the nadir or baseline (PCWG3 criteria) was present in Pros5 and Pros6 (PSA PD). Doce, docetaxel; Caba, cabazitaxel; Mito, mitoxantrone.
